# Crocetinic acid inhibits hedgehog signaling to inhibit pancreatic cancer stem cells

**DOI:** 10.18632/oncotarget.4871

**Published:** 2015-08-13

**Authors:** Parthasarathy Rangarajan, Dharmalingam Subramaniam, Santanu Paul, Deep Kwatra, Kanagaraj Palaniyandi, Shamima Islam, Sitaram Harihar, Satish Ramalingam, William Gutheil, Sandeep Putty, Rohan Pradhan, Subhash Padhye, Danny R. Welch, Shrikant Anant, Animesh Dhar

**Affiliations:** ^1^ Department of Cancer Biology, University of Kansas Medical Center, Kansas City, KS, USA; ^2^ Department of Molecular and Integrative Physiology, University of Kansas Medical Center, Kansas City, KS, USA; ^3^ Department of Pharmaceutical Sciences, University of Missouri at Kansas City, Kansas City, MO, USA; ^4^ Interdisciplinary Science and Technology Research Academy, Abeda Inamdar College, University of Pune, Pune, India

**Keywords:** crocetin, nude mice, pancosphere, DCLK-1, gli transcription factor

## Abstract

Pancreatic cancer is the fourth leading cause of cancer deaths in the US and no significant treatment is currently available. Here, we describe the effect of crocetinic acid, which we purified from commercial saffron compound crocetin using high performance liquid chromatography. Crocetinic acid inhibits proliferation of pancreatic cancer cell lines in a dose- and time-dependent manner. In addition, it induced apoptosis. Moreover, the compound significantly inhibited epidermal growth factor receptor and Akt phosphorylation. Furthermore, crocetinic acid decreased the number and size of the pancospheres in a dose-dependent manner, and suppressed the expression of the marker protein DCLK-1 (Doublecortin Calcium/Calmodulin-Dependent Kinase-1) suggesting that crocetinic acid targets cancer stem cells (CSC). To understand the mechanism of CSC inhibition, the signaling pathways affected by purified crocetinic acid were dissected. Sonic hedgehog (Shh) upon binding to its cognate receptor patched, allows smoothened to accumulate and activate Gli transcription factor. Crocetinic acid inhibited the expression of both Shh and smoothened. Finally, these data were confirmed *in vivo* where the compound at a dose of 0.5 mg/Kg bw suppressed growth of tumor xenografts. Collectively, these data suggest that purified crocetinic acid inhibits pancreatic CSC, thereby inhibiting pancreatic tumorigenesis.

## INTRODUCTION

Pancreatic ductal adenocarcinoma (PDAC) is the 4th leading cause of cancer deaths in developing countries and worldwide, and over 250,000 cases are diagnosed annually. The current standard of treatment is gemcitabine-based combination regimens [1]. However, prognosis remains poor, hence, there is an urgent need for developing more effective therapeutic agents. Commercial Crocetin, obtained from saffron, a spice from the plant *Crocus sativus* L is a carotenoid that has been shown to inhibit nucleic acid and protein synthesis [2, 3]. In previous studies, we demonstrated that commercial crocetin inhibits the growth of pancreatic cancer tumor xenografts [3]. Others have shown that commercial crocetin can affect colon and breast cancer cell lines in culture [3, 4].

Recent evidence suggests the existence of small populations of cancer initiating cells (CSC), which are believed to be responsible for tumor initiation and progression as well as resistance to chemotherapy and radiation. Pancreatic CSC can be allowed to divide and grow in ultra-low binding tissue culture dishes to form multicellular spheroids called pancospheres [5, 6]. Recent studies have identified a rare group of label retaining cancer stem cells that have the capacity to regenerate and also divide and develop tumors. In this regard, it has been shown that the protein Doublecortin Calmodulin-like kinase 1 (DCLK1) marks a morphologically distinct subpopulation of cells with stem cell properties in preinvasive pancreatic cancer [7, 8]. Identification of the regulatory mechanisms and signaling pathways involved in CSC are expected to help identify and design novel agents that target this refractory cell population in PDAC.

Progress related to the mechanism of action has been hampered because commercial or crude crocetin is a mixture of multiple components. In the present study, we report the identification of crocetinic acid, a novel carotenoid molecule that we purified from the commercial crocetin. Furthermore, we demonstrate that purified crocetinic acid significantly suppresses the growth and development of pancreatic ductal adenocarcinoma cell lines in culture and in tumor xenografts.

## RESULTS

### Purification of crocetinic acid

**Figure 1:**
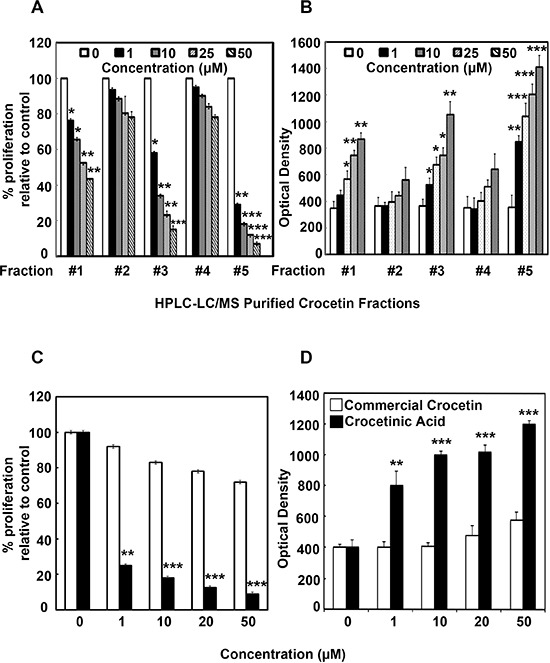
Commercial crocetin and purified crocetinic acid inhibit proliferation and enhance apoptosis Commercial crocetin was obtained and fractionated by HPLC. The fractions were then added to MiaPaCa-2 cells and effect on proliferation assays was determined. **A.** Fractions 1, 3 and 5 have a dose-dependent reduction in proliferation with fraction #5 being the most active. **B.** While all 5 fractions demonstrated dose dependent increase in number of cells undergoing apoptosis, again, fractions 1, 3 and 5 have the most effect. **C.** Comparison of commercial crocetin to purified crocetinic acid, present in fraction #5 shows that doses up to 50 μM, only crocetinic acid is able to inhibit proliferation, while commercial crocetin has no effect. **D.** At doses of less that 50 μM, commercial crocetin does not induce apoptosis, but crocetinic acid is potent and shows a dose dependent increase in apoptosis. *P < 0.05; **P < 0.001, ***P < 0.0001 versus untreated control (Student's *t* test)

Preparative High-performance liquid chromatography (HPLC) was used to fractionate commercial crocetin and Liquid chromatography–mass spectrometry (LC/MS) to characterize the fractions (Supplementary Figure S1). Five major fractions and several minor components were detected (Supplementary Figure S1 panel A). Each fraction was isolated and tested for biological activity. Fraction #5 was further analyzed by LC/MS using ABI 2000 QTrap. A single peak (Supplementary Figure S1, panel B) exhibited the correct mass for crocetin dicarboxylic acid (hereafter designated purified crocetinic acid). H^3^ NMR spectroscopy by both Venyl and Methyl protein analysis suggested that #5 fractions of HPLC is crocetinic acid (Supplementary Figure S1 panel C). Purified crocetinic acid yields from crude crocetin by 0.01 mM sodium hydroxide treatment following HPLC and LC/MS and demonstrated about 50-times more potency in proliferation and apoptosis assays (Figure 1 and Supplementary Figure S2).

### Commercial crocetin and purified crocetinic acid inhibit proliferation and enhance apoptosis

**Figure 2:**
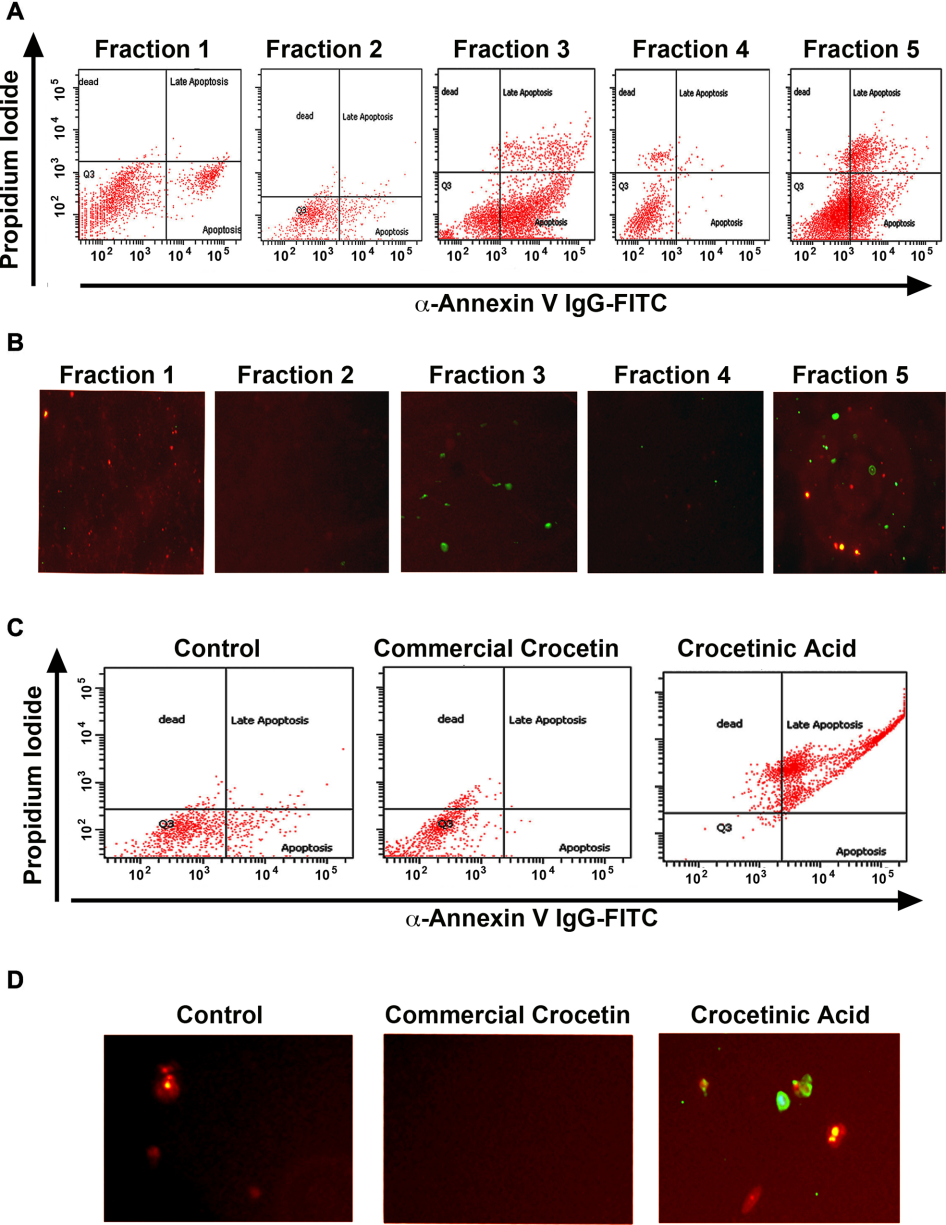
Crocetinic acid induces apoptosis **A, B.** MiaPaCa-2 cells were incubated with 1 μM of the various fraction and levels of apoptosis was determined by flow cytometry (A) and fluorescence microscopy (B). In flow cytometry, propidium iodide staining is for dead cells (Y-axis), while annexin V was determined with a specific antibody conjugated to fluorescein isothiocyanates (FITC). For immunofluorescence, cells treated with FITC were imaged under a fluorescent microscope. Fraction 3 and 5 showed the maximum level of apoptosis by both methods. **C, D.** Panc-1 cells were incubated with 1 μM purified crocetinic acid and commercial crocetin and again tested for apoptosis by flow cytometry and immunofluorescence. Again, there was significantly higher numbers of apoptotic cells in crocetinic acid treated cells.

MiaPaCa-2 cells were treated for 72 h with different fractions derived from commercial crocetin and assessed for proliferation (Figure 1A) and apoptosis (Figure 1B). Fraction 5 significantly reduced proliferation while increasing apoptosis. Subsequently, we compared fraction #5, which contains purified crocetinic acid with commercial crocetin. While purified crocetinic acid significantly reduced proliferation and increased apoptosis of MiaPaCa-2 cells at 1 μM concentration, there was no effect observed with commercial crocetin even up to 50 μM (Figure 1, panel C and D). Similar results were obtained with Panc-1 cells (Supplementary Figure S2). Further confirmation that the compound induces apoptosis was obtained by Annexin5- FITC Flow Cytometry and Fluorescence microscopy (Figure 2). Taken together, these data suggest that fraction #5, which is comprised of crocetinic acid showed 50 times greater effect than commercial crocetin. The remaining fractions, especially fraction #1 and #3 showed little inhibition of proliferation. Therefore, all the mechanistic work has been performed with purified crocetinic acid or HPLC fraction #5 because other HPLC fractions showed little or no effect.

### Status of proliferation and apoptotic signature proteins

Inhibition of proliferation using click-it microplate assay and stimulation of apoptosis microplate assay suggested that purified crocetinic acid is 50 times more potent than commercial crocetin (Figure 1 and Supplementary Figure 2). Even 1 μM purified crocetinic acid showed highly potent inhibition in proliferation and increase in apoptosis than commercial crocetin (Figure 1, 2, Supplementary Figure S2). Inhibition of apoptosis was confirmed by flow cytometry assay and fluorescence microscopy, which also showed significant increase even at lower doses of purified crocetinic acid (Figure 2).

**Figure 3:**
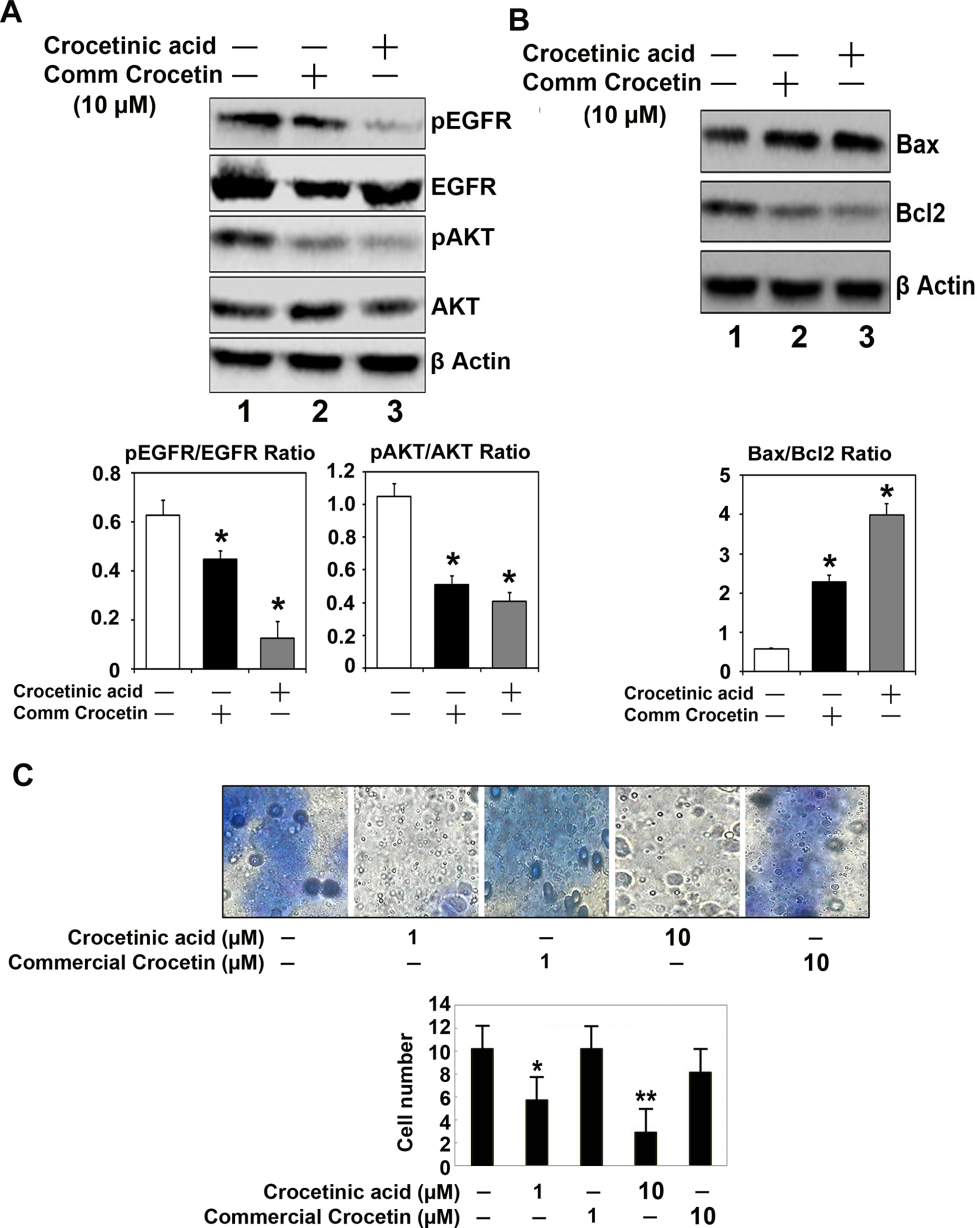
Crocetinic acid inhibits activation of EGF receptor and inhibits cell migration **A.** Effect on EGFR and Akt. MiaPaCa-2 cells were treated with 10 μM commercial crocetin or pure crocetinic acid for 72 h. Extracts were subjected to western blot analyses for phosphorylated and total EGFR and Akt. Crocetinic acid was more potent than commercial crocetin in inhibiting EGFR and Akt phosphorylation. The panel below shows the desitometry readings from three independent experiments. **B.** Effect on Bcl2 and Bax. There was a decrease in Bcl2 expression following treatment with crocetinic acid, and a increase in Bax protein. The graph below shows that the ratio of Bax to Bcl2 is increased significantly following crocetinic acid treatment. **C.** Effects on trans-well migration. MiaPaca-2 cells were seeded in Bowden chamber and allowed to grow and migrate. Commerical crocetin and crocetinic acid was added to the chamber at concentrations of 1 and 10 μM. Purified crocetinic acid significantly inhibited migration of the cells. The graph below shows results from three independent experiments.

Studies have shown that there are at least two types of cells within a cancer cell line, those that are rapidly proliferating progenitor cells and also a rare number of quiescent, cancer stem cells [9]. Epidermal growth factor receptor (EGFR) is a cell-surface tyrosine kinase encoding receptor, which is activated by binding of its specific ligands, including epidermal growth factor and transforming growth factor α (TGFα). EGFR expression and surface localization is significantly upregulated in pancreatic cancers [10]. Upon ligand binding, there is autophosphorylation of its cytoplasmic domains and activation of tyrosine kinase activity occurs, resulting in activation of multiple pathways including the phosphoinositol 3′-kinase/Akt pathway [11]. In turn Akt regulates multiple biological processes including cell proliferation [12]. To determine whether the compound has any effect on proliferating cells, we determined whether phosphorylation of EGFR and AKT are affected. For this, we treated MiaPaCa-2 cells with purified crocetinic acid, and performed western blot analyses for these proteins. As shown in Figure 3, there was significant reduction in the phosphorylation of both EGFR and AKT. We have already shown that the cells undergo apoptosis following treatment with purified crocetinic acid. Hence, we next determined whether there are changes to Bax and Bcl2, since the Bax/Bcl2 ratio is a hallmark of apoptosis. Again, following treatment, there was a significant increase in Bax protein, while that of Bcl2 is significantly reduced, resulting in an increase in the Bax:Bcl2 ratio (Figure 3). Next, we determined whether the migration of the cells is affected after 24 h incubation with the compound using Boyden chamber polycarbonate membranes with 8 micron poresize. MiaPaCa-2 cells/well was loaded into the upper chamber following which they were treated with commercial crocetin and pure crocetinic acid at doses up to 10 μM. The membranes were stained with Giemsa. Five fields of vision per membrane were analyzed using phase-contrast microscopy. Again, the purified crocetinic acid was observed to inhibit migration of cells (Figure 3C).

### Effect of purified crocetinic acid on cancer stem cells

**Figure 4:**
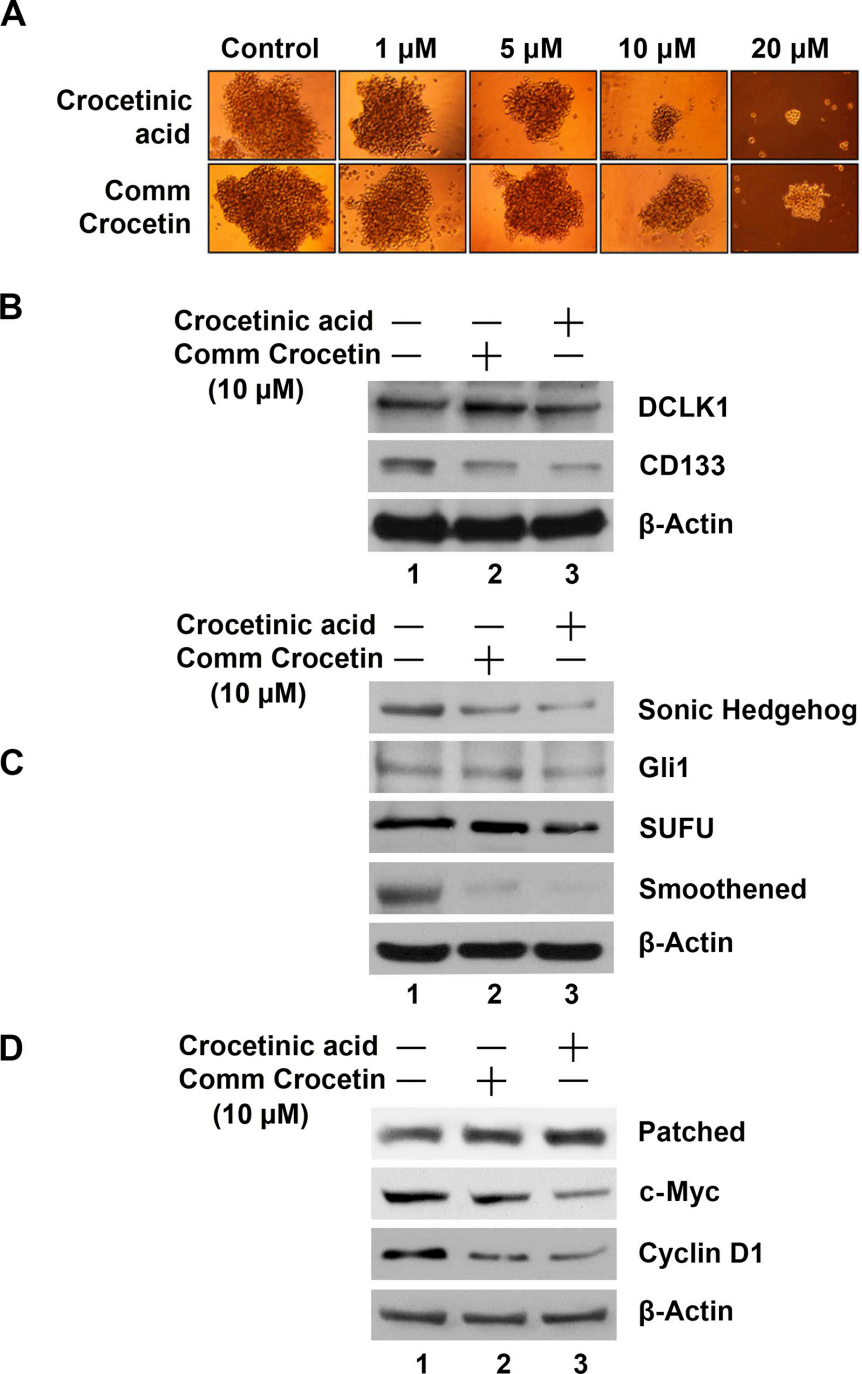
Crocetinic acid inhibits pancosphere formation and Hedgehog signaling pathway **A.** Effect on spheroid formation. MiaPaCa-2 cells plated in ultra-low binding plates were treated with increasing doses (0–20 μM) of commercial crocetin or purified crocetinic acid. Data shows that crocetinic acid is more potent in inhibiting pancosphere formation. **B.** Extracts from MiaPaCa-2 cells were subjected to western blot analyses for various stem cell marker proteins. Data shows that purified crocetinic acid inhibits expression of DCLK1 and CD133. **C, D.** Effects on Sonic Hedgehog signaling pathway. Extracts were also western blotted for various proteins in the hedgehog signaling pathway. Data shows that purified crocetinic acid inhibits the expression of Sonic Hedgehog, the receptor Patched, and signaling related proteins Smoothened, SUFU and transcription factor Gli1. In addition, there was a reduction in targets of the signaling pathway c-Myc and cyclin D1.

Recent evidence suggests the existence of a small population of tumorigenic stem cells responsible for tumor initiation, metastasis and resistance to chemotherapy and radiation. Identification of the regulatory mechanisms and signaling pathways involved in cancer stem cells (CSCs) will help in designing novel agents to target this refractory cell population in pancreatic cancers. Since, we observed that crocetinic acid is able to suppress the proliferation of progenitor cells within the pancreatic cancer cell line, we next determined whether the compound affects cancer stem cells. Sphere-forming assays have been widely used to retrospectively identify stem cells based on their reported capacity to evaluate self-renewal and differentiation. To determine whether crocetinic acid affects pancreatic cancer stem cells, we first performed sphere formation assay. Treatment with purified crocetinic acid decreased the number and size of pancospheres in a dose dependent manner (Figure 4A). Moreover, there was also a significant decrease in the size of secondary and tertiary spheroids in the crocetinic acid treated samples (data not shown), suggesting that the purified compound targets CSCs. To further confirm that the stem cells are affected by crocetinic acid, western blot analyses was performed for stem cell marker proteins. Treatment with crocetinic acid significantly suppressed the expression of CD133 and DCLK1 (Figure 4B). Taken together, these data suggest that the compound has significant effects on pancreatic cancer stem cells.

Next, to further understand the mechanism of inhibition of the stem cells, we determined which signaling pathways are affected. Multiple signaling pathways are known to be important for stemness, including the Wnt/Δ-catenin, Notch and Sonic Hedgehog (SHH) pathways [13]. Preliminary studies suggested that the SHH/Gli pathway is significantly affected following treatment with purified crocetinic acid. The SHH/Gli pathway is known to play a significant role in developmental and cancer biology [14]. The SHH pathway is essential during development for pattern formation [15]. Moreover, in the adult tissues, the pathway affects transcription of genes involved in cell homeostasis [16]. More importantly, in relation to cancer, the SHH/Gli pathway is known to promote self-renewal of CSCs by transcriptionally regulating expression of various genes [17]. Hedgehog signaling is initiated by the binding of hedgehog protein to its cognate receptor Patched on the cell surface [18]. This results in the derepression of Smoothened (Smo) protein, which in turn mediates downstream signal transduction, and finally dissociating the Gli proteins from a key intracellular Hh pathway regulator SUFU. The Gli protein is a transcription factor, which when released from the complex can translocate to the nucleus, where it binds to its cognate DNA binding site in the promoters of target genes such as c-Myc and cyclin D1, and induces their expression. To confirm that the compound inhibits hedgehog signaling pathway, we performed western blot analyses for proteins involved in the pathway including SHH, its receptor Patched and the intracellular signaling regulators Smoothened and SUFU, the transcription factor Gli1, and a Hh target gene cyclin D1 and c-Myc. Treatment with crude crocetin showed a relatively modest effect in the expression of all these proteins (Figure 4C, 4D). However, when cells were exposed to crocetinic acid, there was significant suppression in the expression of these Hh-signaling proteins. These data suggest that crocetinic acid affects pancreatic cancer stem cells in part through suppression of the hedgehog-signaling pathway.

### Crocetinic acid suppresses tumor xenograft growth

**Figure 5:**
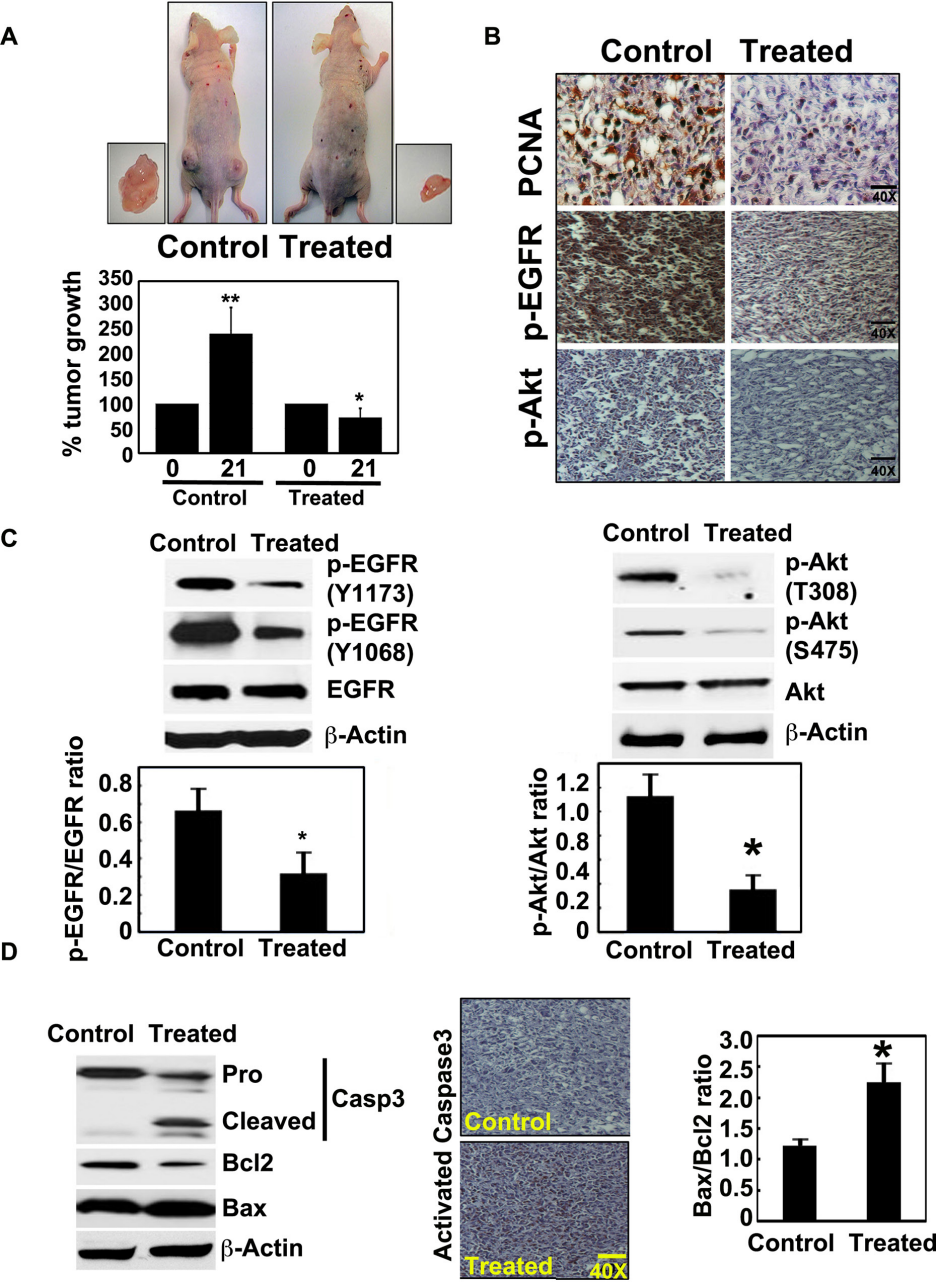
Crocetinic acid inhibits pancreatic cancer xenografts **A.** Xenograft tumor growth in athymic (nude) mice. Tumor growth is significantly inhibited after purified crocetinic acid was administered at a dose of 0.5 mg/kg. The tumor size was measured from the start date of crocetin treatment (0 day) to end date (21 days). Data shows that there was significant inhibiton of the growth of the tumors in the treated group. **B.** Immunohistochemistry analysis of the xenograft tissues show that there is a significant reduction in levels of PCNA, and phosphorylated EGFR and Akt proteins. **C.** Western Blot analyses of extracts from the xenograft tissues also demonstrate reduced levels of phophorylated EGFR and phophorylated Akt. Ratios of phosphorylated proteins with total proteins show significant reduction in phosphorylation of both proteins. **D.** Crocetinic acid induces apoptosis in tumor xenograft tissues. Western blot analyses shows significant activation/cleavage of the effector capsase in the apoptosis pathway, caspase 3. In addition, there was a reduction in Bcl2 protein expression while there was an induction of Bax protein. Immunohistochemistry also revealed significant increase in activated capase 3 in the crocentinic acid treated mice. *P < 0.05; **P < 0.001 versus untreated control (Student's *t* test).

We next determined the effect of purified crocetinic acid on pancreatic cancer xenografts. For this, we used MiaPaCa-2 cells because these cells are characteristically more aggressive than most other pancreatic cancer cells and have the ability to develop tumors within several weeks of inoculation [9]. MiaPaCa-2 cells were inoculated into the flanks of athymic nude-mice, and tumors were allowed to develop (about 3 weeks). Then, the mice were treated with purified crocetinic acid (0.5 mg/kg/day) for 30 days. The tumors were measured twice per week until the mice were euthanized. Tumor incidence was 100% in all the animals. However, the tumors in the treated group were significantly smaller than in the untreated controls (Figure 5A).

We next confirmed the results of our *in vitro* studies with isolated tumor xenograft tissues. To determine whether the regression in tumor growth by purified crocetinic acid is due to inhibition of proliferation, apoptotic cell death or both, we first determined the expression of proliferating cell nuclear antigen (PCNA) as well as that of phosphorylation of EGFR and Akt. Immunohistochemistry analyses demonstrated significantly reduced levels of PCNA positive cells, and that of phosphorylated EGFR and Akt in xenograft tissues obtained from animals that were administered purified crocetinic acid, when compared to controls (Figure 5B). We confirmed the reduction in phosphorylation by western blot analyses. For EGFR, we looked at specific phosphorylation at tyrosine residues at 1168 and 1173. Phosphorylation of EGFR at both sites was significantly suppressed in the tissues of crocetinic acid-treated animals (Figure 5C). Additionally, EGFR activity as determined by the ratio of EGFR phosphorylated and unphosphorylated form was significantly impaired in the tumors due to purified crocetinic acid treatment (Figure 5C). Similarly, we determined the effect of treatment on phosphorylation of Akt at threonine 308 and serine 475. Again, it was significantly reduced in tissues from treated animals (Figure 5C). Next, we determined whether the cells were undergoing apoptosis. Western blot analyses demonstrated significant increase in cleaved caspase 3 in crocetinic acid-treated animals, which was further confirmed by immunohistochemistry (Figure 5D). To further confirm the apoptotic effect, the Bax/Bcl2 ratio was evaluated. As shown in Figure 5D, expression of Bax protein was increased with a concomitant decrease of Bcl2 protein. Collectively these sets of experiments indicate that inhibition of tumor growth is due to the induction of apoptosis as well as inhibition of proliferation.

## DISCUSSION

Pancreatic cancer is the fifth leading cause of cancer death in the Western world [1, 6]. Pancreatic tumors are highly resistant to current available therapies and the 5-year survival is dismal with a median expected post-diagnosis survival time of five months [1, 6, 19]. Owing to poor prognosis, alternative therapies are being investigated. Crocetin, a carotenoid compound derived from saffron, has demonstrated a significant inhibitory effect on the growth of cancer cells [3, 4]. Potential mechanisms for crocetin mediated inhibition of tumor growth include the reduction in the synthesis of DNA, RNA and protein [20, 21]. It has also been demonstrated that crocetin inhibits RNA polymerase II activity [4], [20]. Crocetin also interferes with histone H1 structure and H1-DNA interactions suggesting for another possible mechanism of anticarcinogenic action [22]. The exact mechanism of the protective activity of crocetin is not clear at present but several hypotheses have been forwarded which suggest that carotenoids can convert to vitamin A, can enhance carcinogen metabolism, can act as an antioxidant or can inhibit nucleic acid synthesis [3, 4]. However, as we were performing the studies, we realized that commercially available crocetin is really made up of multiple compounds. In the present study, we have separated novel crocetin compounds using HPLC and LC/MS. Five fractions were separated from commercial crocetin using HPLC of which 5^th^ fraction (purified crocetinic acid) showed most effective inhibition of proliferation and inducer of apoptosis. Similarly, pancreatic cancer growth in nude mice was also significantly inhibited due to the oral administration with purified crocetinic acid. Therefore, it was imperative to investigate the effect of the novel purified crocetinic acid on the proliferation of pancreatic cancer cells and pancreatic tumor regression in nude mice.

The purified crocetinic acid demonstrated more pronounced effect on proliferation and apoptosis even at concentrations less than 10 μM, which is signifcantly lower than that seen with commercially available crocetin. While there was some effect with other fractions, they were not as potent as crocetinic acid, the only compound found in HPLC fraction 5. Hence, although we have focused our work in this manuscript on crocetinic acid, there are probably other less potent compounds present in these other fractions that may also merit from further studies.

EGFR is a critical regulator of cellular proliferation and differentiation and plays a central role in tumor proliferation and growth [23–25]. Purified crocetinic acid significantly reduced EGFR activity in pancreatic cancer cells. Immunohistochemical and western blot analysis also revealed a significant decrease of EGFR phosphorylation in the xenograft tumors from crocetinic acid treated mice. It is also important to note that EGFR phosphorylation reduces PCNA levels inside the nucleus, which is essential for DNA replication [26]. Moreover, it has been demonstrated that EGFR phosphorylation activated Akt signaling pathways in fostering proliferation and survival [27]. Therefore, inhibition of phosphorylation of EGFR by crocetinic acid interrupts phophorylation of Akt, which in turn interferes with tumorigenesis. These studies indicate that purified crocetinic acid is an effective inhibitor of EGFR activity and this inhibition correlates with impaired tumor xenograft growth.

Previously, we have shown that the commercially available crocetin induces cytotoxicity of tumor cells [3, 4]. Here, we have shown that purified crocetinic acid induces apoptosis or programmed cell death. Members of the Bcl2 family of proteins have been shown to play a significant role in controlling apoptosis. These proteins govern mitochondrial outer membrane permeabilization and can be either pro-apoptotic or anti-apoptotic. Bcl2 itself is an antiapoptotic protein that can be induced by a variety of physiological and pathological stimuli. On the other hand, Bax has a pro-apoptotic effect and also counters anti-apoptotic effect of Bcl2 [28, 29]. It has been proposed that the ratio of Bax/Bcl2 may govern the sensitivity of cells of apoptotic stimuli [30]. In this study, the ratio of Bax/Bcl2 was significantly increased in both pancreatic cancer cells as well as in pancreatic tumors in response to treatment with purified crocetinic acid, which suggests that crocetinic acid modulates mitochondrial function to mediate cell death.

It is now widely believed that long-lived, rare cells, termed stem cells present in both tissues and in cancers have the ability to self–renew and regenerate. Self-renewal is one of the defining characteristics of stem cells, results in daughter cells remaining undifferentiated, retaining the ability to give rise to another stem cell [31, 32]. A hallmark feature of cancer stem cells is the formation of large, floating spheres, termed spheroids that can be serially passaged [33]. These spheres are highly tumorigenic and capable of forming colonies *in vitro*. The CSC theory asserts that many types of cancer are initiated from and maintained by a minor population of tumorigenic cells that are capable of continuous self-renewal and differentiation [34, 35]. This cell population undergoes unlimited proliferation and gives rise to differentiated cells, developing new tumors phenotypically recapitulating the original tumors [7]. Here, we have demonstrated that treatment of pancreatic spheroids or pancospheres with purified crocetinic acid resulted in significant inhibition of the spheroids. This suggests that the compound has the ability to impair CSC self-renewal. Furthermore, we have shown that the compound significantly suppresses the expression of Doublecortin and Ca2^+^/calmodulin-dependent kinase-like-1 (DCLK1) and Promenin/CD133. We have previously demonstrated that DCLK1 is a stem cell marker for both normal pancreatic cells and pancreatic cancer tissues. This has also recently been confirmed by more recent studies by Leach and colleagues [7, 8]. Since stem cells were affected, we tuned towards potential stem cell pathways. In this regard, we look at the hedgehog signaling pathway, which is involved in self-renewal of CSC. Hedgehog-Gli signaling has also been shown to control the self-renewal behavior of human glioma CSCs and tumorigenicity [36]. Hedgehog signals through binding to its transmembrane receptor, Patched (Ptch). In the absence of hedgehog ligands (which includes Sonic Hedgehog, Indian Hedgehog and Desert Hedgehog), Ptch associates with Smoothened (Smo) and blocks Smo function [36]. When hedgehog (HH) binds to Ptch, Smo is released, triggering dissociation of transcription factors, Gli1 and suppressor of Fused (SuFu), leading to transcription of an array of genes, such as cyclin D1 [35, 36]. Here, we observed that following treatment with purified crocetinic acid there was significantly lower levels of Smo while Ptch expression is increased. Furthermore, there were significantly reduced levels of Gli1 and the downstream target genes for the pathway cyclinD1 and c-Myc. The mechanism by which Gli levels are reduced is currently unknown, because this could be due to lack of Gli1 gene transcription or increased degradation of the protein. Also, how this affects the overall signaling pathways, and what cross talk exists between this and other signaling pathways that are affected by crocetinic acid to inhibit the expression of either cyclin D1 or c-Myc would be interesting to determine because other pathways can also induce expression of these same genes. In this regard, there is cross talk between the hedgehog signaling pathways and EGFR signaling pathways and that EGFR signaling is key step for hedgehog signaling in human malignancies. A growing body of evidence also suggest that Gli activation could be modulated via EGFR/PI3Kinase/Akt signaling pathways because this can directly affect Gli expression [37, 38]. Therefore, inhibition of EGFR/Akt signaling pathways by purified crocetinic acid may be one reason for the significant impact in hedgehog signaling and stemness of pancreatic cancer progression.

**Figure 6:**
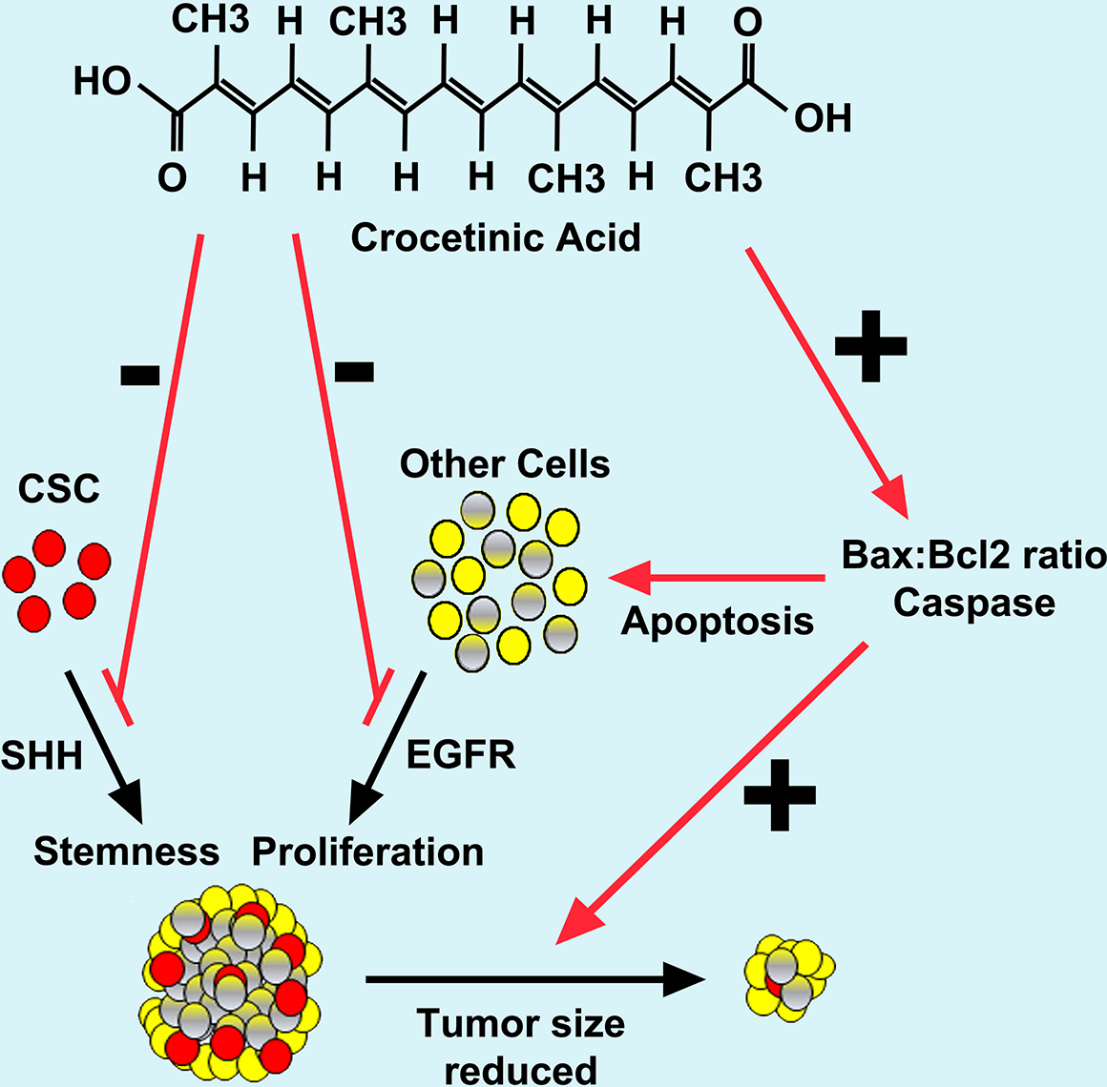
Schematic representation of mechanism by which crocetinic acid inhibits tumor growth

The fact that crocetinic acid was effective in inhibiting pancreatic cancer cells from proliferating and develops tumor xenografts is promising and suggests the need to further investigate its utility for therapy and preventive purposes. The dosages of purified crocetin used in the *in vivo* studies are also within acceptable range of dosing as suggested by other investigators [3], [23, 39]. It is also noteworthy that we were able to administer purified crocetin for extended period of time. One thing to note is that the dose of crocetinic acid used in this study would be considered as pharmacological therapeutic doses and hence the compound should be treated as a chemotherapeutic agent. In this regard, a major problem with anticancer agents is their toxic effect on normal cells. The concentration of purified crocetinic acid used in this study appears to not be toxic to human cells. Previous studies have also reported the LD_50_ of crocetin to be 2g/kg [39, 40]. It has also been suggested that carotenoids are well tolerated at high doses and numerous studies have supported their use in cancer chemoprevention and chemotherapy [41]. In a similar fashion, crocetinic acid appears to also be relatively non-toxic. However, detailed bioavailability and pharmacokinetic and pharmacodynamics studies are necessary to ensure that the compound is safe. Also, currently all we know is that the compound does not allow tumor growth, with the xenograft study being a tumor growth delay. In summary, our study demonstrated that crocetinic acid down-regulated proliferation by targeting CSC and stimulated apoptosis that resulted in significant growth regression in both *in vitro* pancreatic cancer cells and *in vivo* pancreatic tumors (Figure 6). What would be interesting to determine is whether the compound is also effective in pancreatic cancer regression, and whether the mechanism of tumor regression is similar to that of inhibiting tumor growth.

In conclusion, this study indicates for the first time that purified crocetinic acid could be used as a novel therapeutic agent for pancreatic cancer. Further studies, as mentioned above related to dosing and formulations would greatly benefit moving the compound to the clinic to determine therapeutic efficacy in humans affected by pancreatic and other cancers.

## MATERIALS AND METHODS

### Reagents

Commercial crocetin and antibodies to phosphorylated Akt and EGFR were obtained from Cell Signaling (Danvers, MA), EGFR from BD Biosciences (San Jose, CA), Bcl2 from Calbiochem (San Diego, CA), Bax polyclonal antibodies from Santa Cruz Biotechnology (Santa Cruz, CA), and β-actin from Sigma Chemical Co. (St. Louis, MO). The FITC Annexin V/Dead Cell Apoptosis assay was purchased from Invitrogen (Life Technologies, Green Island, NY). Click-it EdU microplate proliferation assay was purchased from Invitrogen (Life Technologies, Green Island, NY). Peptamen was purchased from Nestle (Los Angeles, CA).

### Cell lines, culture conditions and treatment

Human PDAC cell lines MiaPaCa-2, BxPC-3, Capan-1 and ASPC-1 were obtained from the American Type Culture Collection (ATCC, Manassas, VA, USA) and grown in Dulbecco's modified Eagles medium (DMEM, Sigma Chemical Co, St Louis, MO) supplemented with 1 mM sodium pyruvate (Fisher Chemical Company), 100 U/ml of penicillin and 100 U/ml of streptomycin (Sigma Chemical Co, St Louis, MO) and 10% FBS (Hyclone, Road Logan, UT) at 37°C in a humidified atmosphere containing 5% CO_2_. Semi-confluent (~70%) cells were treated with various concentrations (1– 50 μM) of purified or commercial crocetin as indicated.

### Purification of crocetinic acid

Commercial crocetin contains at least 5 major components and several minor components (Supplementary Figure 1). Commercial crocetin was purified by preparative HPLC on an Agilent 1050 series HPLC equipped with diode array detector and Gilson fraction collector. Chromatographic elution was performed using an Alltech Econosphere C18 column (250 × 10 mm, 10 *μ*m particles) at a flow rate of 2 mL/min for the initial 4 min and then 3 mL/min thereafter, with a gradient of 100% solvent A (0.1% Formic acid in water) for 4 min, then 0% to 100% of solvent B (methanol) in next 60 min, then a linear gradient of 100% solvent B for the next 10 min, then 0% to 100% of solvent A in next 1 min, and then 100% solvent A for the next 15 min. For preparative HPLC, commercial crocetin was fractionated on an Agilent 1050 series. Each fraction was collected and characterized for biological activity using proliferation and apoptosis assays.

### LC/MS

LC/MS analysis was carried out on the HPLC collected fraction of commercial crocetin using ABI 2000 QTrap. Detection of ions was performed in EMS positive mode. Commercial crocetin fraction of HPLC demonstrated a single peak in LC/MS with the correct mass for crocetin dicarboxylic acid (purified crocetinic acid). In addition, Varian Inova 400 MHz NMR system was used for purified #5 fractions or purified crocetin separated from Commercial crocetin

### Proliferation assay

Pancreatic cancer cells were plated at desired density onto 96-well Corning tissue culture plates. After 24 hours, cells were treated for 72 h with various concentrations (1–50 μM) of purified crocetinic acid and commercial crocetin. Cells were labeled with Click-it EdU and fixed, incubated with anti-Oregon Green antibody conjugated to horseradish peroxidase and fluorescent product measured by spectrophotometry (excitation/emission~568/585).

### Apoptosis assay

Cells (MiaPaCa-2 and Panc-1) were treated with different concentrations (1–50 μM) of purified crocetin and commercial crocetin and DNA fragmentation was measured using modified TUNEL (TdT-mediated dUTP Nick-End Labeling) assay (Dead End Colorimetric TUNEL assay from Promega) per manufacturer's instructions. FITC Annexin V and propidium iodide staining and flow cytometry provide a rapid and convenient assay for apoptosis. Propidium iodide stains necrotic cells with red fluorescence whereas green FITC dye stains apoptotic cells.

### Trans-well migration assay

Migration of MiaPaCa-2 cells was done as previously described [3]. Briefly, cells were seeded in Boyden chambers, incubated for 24–72 h, stained with Giemsa and cells traversing the membrane were counted.

### Tumor xenograft studies

Six-eight week old athymic female mice were obtained from Charles River Laboratories, (Wilmington, MA). All animals were maintained in an aseptic environment with 12-hr light/dark cycle in food and water was provided. All procedures were reviewed and monitored by the KUMC Institutional Animal Care and Use Committee. MiaPaCa-2 cells (2.5 × 10^6^) was injected subcutaneously into the flanks of each mice and tumors were allowed to form. Once palpable tumors developed (~0.34 cm^3^), mice were randomly divided into two groups (6 mice per group), and purified crocetinic acid dissolved in 15 ml of peptamen was administered orally during the dark cycle for 30 days. The drug concentration administered (~0.5 mg/kg/day) is in agreement with other dose ranges published [3], [19]. The control mice were treated with only peptamen (i.e., vehicle). During the day time, mice were given solid food for 12 hours. Tumor size was monitored twice weekly, and its volume calculated as V = (a × b)^2^/2, where a = length and b = width. All mice were euthanized after 30 days, and tumors were removed, measured, flash frozen and stored at −80°C.

### Western blot analysis

Immunoblotting was performed as previously described [3]. Briefly, MiaPaCa-2 and Panc-1 cells or tumors with or without purified crocetinic acid treatment were homogenized, and the lysates centrifuged at 18000 × g, 1 hr, at 4°C to precipitate the particulates. The supernatant was collected and used for immunodetection.

### Immunohistochemistry

Immunohistochemical analysis was performed according to our previously published method [3] using Zymed broad range immunohistochemical kit. Briefly, tumor tissue samples were fixed in 4% neutral buffered formalin and embedded in paraffin. The serial sections (5 μm) were deparaffinized and re-hydrated in graded ethanol solutions. Endogenous peroxidase activity was blocked at room temperature by incubating in hydrogen peroxide in methanol (1:9). Slides were washed with PBS and nonspecific binding was blocked by blocking solution for 10 min. The sections were incubated with respective primary antibody in a humidified chamber at 4°C overnight. The slides were incubated with biotinylated secondary antibody (Zymed Laboratories, CA) then 3,3′-diaminobenzidine (DAB) before counter staining with haematoxylin.

### Pancosphere assay

Spheroids were prepared as previously described [5]. Briefly, 1000 cells/well in DMEM/F12 (Invitrogen, France) supplemented with EGF (20 ng/ml, Invitrogen) and B27 supplement (1x, Invitrogen) were distributed in in ultra-low attachment 6 well plates. Plates were placed in a humidified atmosphere of 5% CO2 at 37°C. Numbers of pancospheres after 6–8 days culture were counted under a light microscopy and quantified by Celigo.

### Statistical analysis

All experiments included triplicate samples for each of the observation. Each experiment was repeated three times. Data are presented as mean ± standard deviation experiments from consolidated. Statistical analysis was performed between the groups of data by paired Student's *t*-test. *P*-value less than 0.05 were considered as statistically significant.

## SUPPLEMENTARY FIGURES AND LEGENDS


