# Supplementary material for: Crocetinic acid inhibits hedgehog signaling to inhibit pancreatic cancer stem cells
Source: Oncotarget. 2015 Aug 13;6(29):27661–73. doi: 10.18632/oncotarget.4871 (PMC4695016; doi:10.18632/oncotarget.4871)
Supplement: Supplementary file 1 [file oncotarget-06-27661-s001.pdf]

SUPPLEMENTARY FIGURES AND LEGENDS

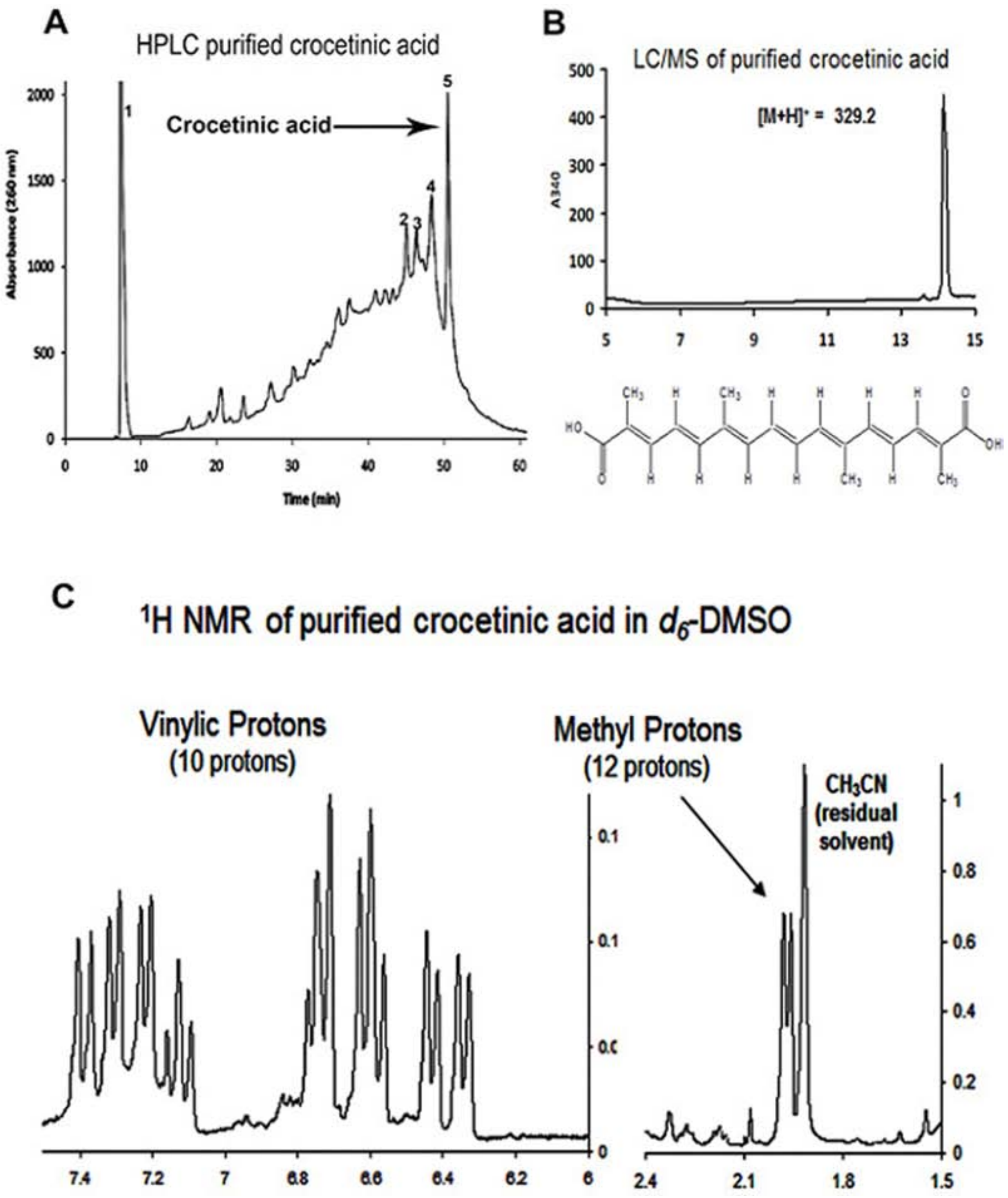

Supplementary Figure S1: Supplementary 1**A.** HPLC Purification of Crocetinic Acid from Commercial Crocetin: Commercial crocetin was subjected to preparative HPLC on Agilent 1050 series HPLC equipped with diode array detector. Fractions were collected in Gilson fraction collector. Fraction 5 is purified crocetinic acid. Supplementary 1**B**. LC/MS of Purified Crocetinic Acid eluted from HPLC: LC/MS analysis was carried out on the fractions collected, using ABI 2000 QTrap with an electron spray ionization(ESI) source, interfaced to an Agilent 1100 series HPLC system equipped with diode array detector. Supplementary 1**C**. NMR Spectroscopy: H3 NMR spectroscopy analysis was carried though Vinyl and Methyl protons of HPLC purified #5 fractions or PC.

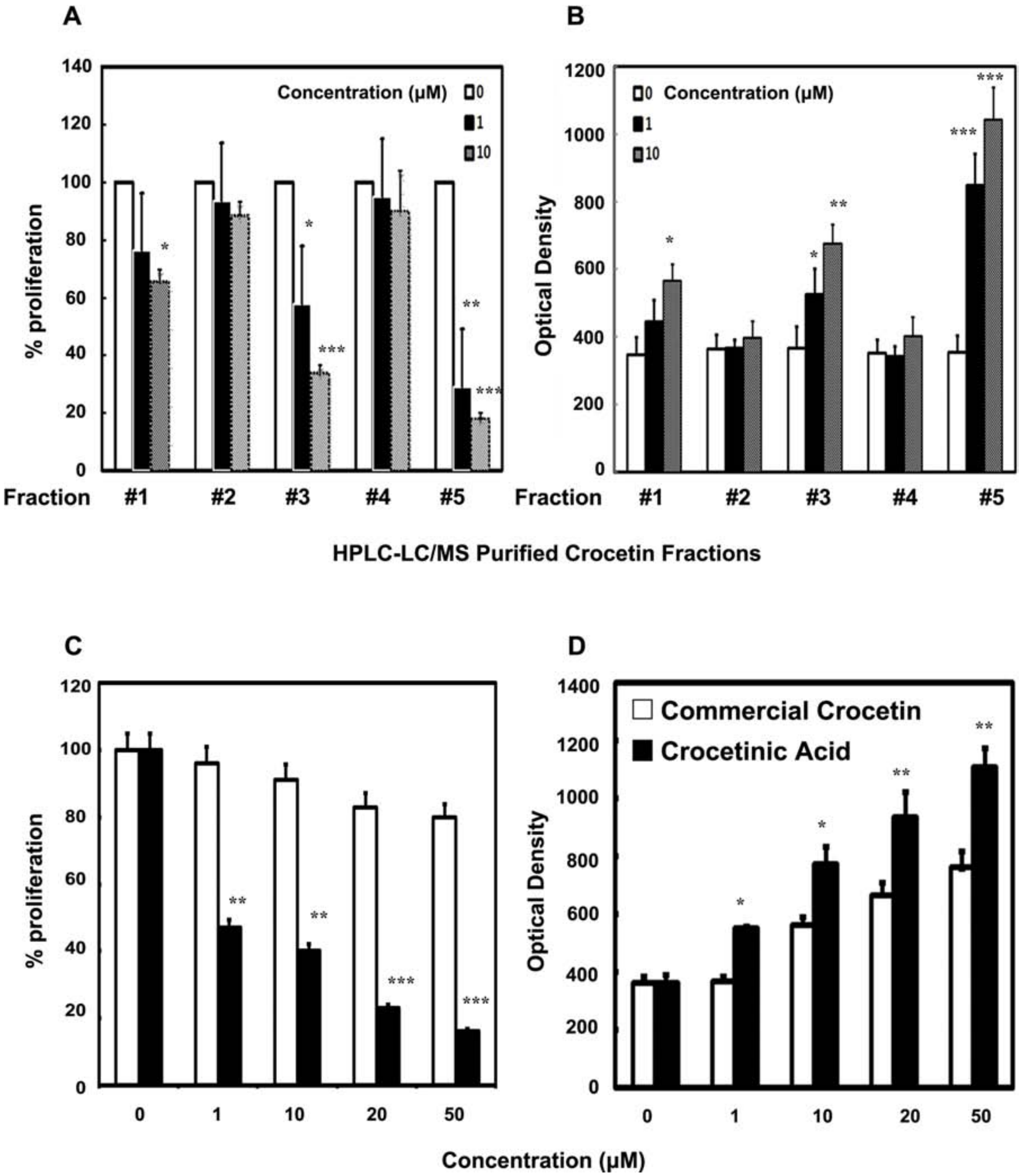

Supplementary Figure S2: Different purified HPLC fractions were tested on Panc-1 cells at increasing doses (0–10 µM). **A.** Fractions 1, 3 and 5 have a dose-dependent reduction in proliferation with fraction being the most active. **B.** While all 5 fractions demonstrated dose dependent increase in number of cells undergoing apoptosis, again, fractions 1, 3 and 5 have the most effect. **C.** Comparison of commercial crocetin to purified crocetinic acid, present in fraction #5 shows that doses up to 50 µM, only crocetinic acid is able to inhibit proliferation, while commercial crocetin has no effect. **D.** At doses of less that 50 µM, commercial crocetin does not induce apoptosis, but crocetinic acid is potent and shows a dose dependent increase in apoptosis. *$P < 0.05$; **$P < 0.001$, ***$P < 0.0001$ versus untreated control (Student's *t* test).
